# It's a risky business: use of the QCovid risk calculator in a psychiatric rehabilitation population to enhance prevention

**DOI:** 10.1192/bjo.2021.172

**Published:** 2021-06-18

**Authors:** Jinal Patel, Fraser Scott, Rajesh Mohan

**Affiliations:** Heather Close Rehabilitation Unit, South London and Maudsley NHS Foundation trust

## Abstract

**Aims:**

Serious mental illness (SMI) is now accepted as a significant risk factor for contracting COVID-19, increasing the rates of adverse outcomes, including hospitalisation and mortality. Risk assessments are the cornerstone of protecting vulnerable groups of individuals. The QCovid risk calculator is a newly developed tool to predict the risk of death or hospitalisation from COVID-19. It has not been applied in SMI populations. We aimed to use the QCovid risk calculator in an inpatient rehabilitation setting to identify and mitigate risk for people with SMI with personalised COVID-19 prevention plans.

**Method:**

Clinical and sociodemographic characteristics were obtained for 22 inpatients. Firstly, the QCovid risk calculator was used to ascertain the absolute and relative risks to patients (Odds Ratios (OR) of mortality and/or hospitalisation) from COVID-19. Patients were stratified as high (OR > 10), moderate (OR 5-10) and low (OR < 5) risk. Secondly, personalised COVID-19 prevention plans were coproduced by patients and clinicians addressing 1) risk factors contributing to increased QCovid risk, 2) patient's personal goals, concerns, and preferences 3) maximizing patient engagement in COVID-19 infection prevention strategies. Finally, uptake of personalised COVID-19 prevention plans was evaluated after four weeks using a customised patient feedback questionnaire.

**Result:**

Of the 22 inpatients (68% male), 14 patients (64%) had schizophrenia and 3 patients (14%) had schizoaffective disorder as primary diagnosis. 13 (59%) patients were prescribed clozapine. QCovid risk stratification showed 10% of patients as high risk, 29% as moderate risk, and 61% as low risk. Apart from SMI in all 22 inpatients, the most common QCovid risk factors were increased body mass index (64%, n = 14; 23% overweight and 41% obese), diabetes mellitus type 1 or 2 (27%, n = 6) and epilepsy (n = 4, 18%). 19 of the 22 patients provided feedback on their personalised COVID-19 prevention plans. Most patients (79%) felt they had “contributed significantly” to their COVID-19 prevention plans, and their individual goals and concerns were valued. 79% were “satisfied” with their COVID-19 prevention plans. Subjective perception of safety from COVID-19 was high, with 95% of patients feeling “safe and well-protected from COVID-19”.

**Conclusion:**

Comprehensive assessment of COVID-19 risks in vulnerable groups enables personalised risk mitigation, both at an individual and service level. Our findings show the importance of applying current knowledge to protect vulnerable patients with SMI through personalised prevention plans. This approach can be scaled up to understand risks for services and teams, while allowing clinicians to adapt their use for individualised COVID-19 prevention.

